# Development of a chemogenomics library for phenotypic screening

**DOI:** 10.1186/s13321-021-00569-1

**Published:** 2021-11-24

**Authors:** Bryan Dafniet, Natacha Cerisier, Batiste Boezio, Anaelle Clary, Pierre Ducrot, Thierry Dorval, Arnaud Gohier, David Brown, Karine Audouze, Olivier Taboureau

**Affiliations:** 1 Université de Paris, INSERM U1133, CNRS UMR8251, 75006 Paris, France; 2 Institut de Recherche Servier, 125 Chemin de Ronde, 78290 Croissy-sur-Seine, France; 3 Université de Paris, INSERM UMR S-1124, 75006 Paris, France

**Keywords:** Phenotypic screening, Phenotypic drug discovery, Chemical biology, System pharmacology network, Network pharmacology, Chemogenomics

## Abstract

With the development of advanced technologies in cell-based phenotypic screening, phenotypic drug discovery (PDD) strategies have re-emerged as promising approaches in the identification and development of novel and safe drugs. However, phenotypic screening does not rely on knowledge of specific drug targets and needs to be combined with chemical biology approaches to identify therapeutic targets and mechanisms of actions induced by drugs and associated with an observable phenotype. In this study, we developed a system pharmacology network integrating drug-target-pathway-disease relationships as well as morphological profile from an existing high content imaging-based high-throughput phenotypic profiling assay known as “Cell Painting”. Furthermore, from this network, a chemogenomic library of 5000 small molecules that represent a large and diverse panel of drug targets involved in diverse biological effects and diseases has been developed. Such a platform and a chemogenomic library could assist in the target identification and mechanism deconvolution of some phenotypic assays. The usefulness of the platform is illustrated through examples.

**Supplementary Information:**

The online version contains supplementary material available at 10.1186/s13321-021-00569-1.

## Introduction

In the past 2 decades, the drug discovery paradigm has shifted from a reductionist vision (one target—one drug) to a more complex systems pharmacology perspective (one drug—several targets) [1]. The reasons are related, notably, to the number of failures of drug candidates in advanced stages of clinical trials due to a lack of efficacy and clinical safety [2]. Furthermore, the traditional expectations that selective ligands act on a single target are now challenged with new drug discovery processes, especially for complex diseases like cancers, neurological disorders and diabetes as they are often caused by multiple molecular abnormalities rather than being the result of a single defect [3–5].

To accelerate drug discovery research in chemogenomic, systematic screening programmes of targeted chemical libraries against a set of protein families have emerged. For example, to discover new drugs to treat cancer, a library consisting of known kinase inhibitors may be screened to identify hit compounds and then start a medicinal chemistry programme. Similar exercises have been performed with GPCR-focused libraries [6] and protein–protein interaction inhibitors [7].

More general chemical libraries were also built up representing collections of selective small pharmacological molecules that can modulate protein’s targets across the human proteome and be involved in a phenotype perturbation. With the increased facility for academics to get access to large chemical libraries, chemogenomic, proteochemometric or polypharmacology approaches have started to be developed allowing to mine this vast amount of protein–ligand interactions and to predict a single ligand against a set of heterogeneous targets [8–10]. Associations between drug-target and gene-disease started to be investigated through druggable genome studies [11–13]. Collection and processing of a wide array of genomic, proteomic, chemical and disease-related resource data were also explored using network pharmacology approaches [14, 15]. Network pharmacology combines network sciences and chemical biology allowing the integration of heterogeneous sources of data and the possibility to look over the action of a drug on several protein targets and their related biological regulatory processes in system biology [16]. Multiple studies have reported new insights in drug target clinical outcomes based on the combination of chemogenomics, network analysis and diseases [17–19].

Among chemical libraries considered in chemogenomic studies, many of them have been built by industrial companies like the Pfizer chemogenomic library, the GlaxoSmithKline (GSK) Biologically Diverse Compound Set (BDCS), Prestwick Chemical Library and the Sigma-Aldrich Library of Pharmacologically Active Compounds, but some of them are also available for public screening programmes like the Mechanism Interrogation PlatE (MIPE) library that was developed by the National Center for Advancing Translational Sciences (NCATS). More details about these chemogenomics libraries can be found here [20].

For a few years, there has been a revival of phenotypic screening in drug discovery. However, the chemical libraries discussed previously are not always optimised for such studies. In fact, with the advances in various technologies for cell-based phenotypic screening, including the development of induced pluripotent stem (iPS) cell technologies, gene-editing tools such as CRISPR-Cas and imaging assays technologies, new phenotypic drug discovery studies are reported in the literature [21–25]. Image-based high-content screening (HCS) on 30,000 small molecules has been for example used with a generative adversarial network to propose new small molecule structures that share similar morphological profile [25]. Therefore, as phenotypic drug discovery studies do not rely on knowledge of the molecular target perturbed by a specific drug, the translation of the molecular mechanism of action in the context of a disease-relevant cell system i.e., molecular phenotyping is the next challenge.

In this context, we decided to develop a pharmacology network for phenotypic screening, integrating the ChEMBL database [26], pathways, diseases and a high-content image-based assay for morphological profiling, Cell Painting [27], in a high‐performance NoSQL graphics database (Neo4j^®^). The aim is to identify proteins modulated by chemicals that could be related to some morphological perturbations at the cell level and lead to some phenotypes, diseases and/or adverse outcomes. Furthermore, a chemogenomic library of 5000 small molecules that represents a large panel of drug targets involved in diverse biological effects and diseases was built. Using filtering based on scaffolds, this library encompasses the druggable genome represented within our network pharmacology and that can be of interest for phenotypic screening. The protocol considered in the development of the network pharmacology is discussed further through examples in the next sections.

## Materials and methods

### Database

#### ChEMBL

The ChEMBL database (version 22) [28] was used for this analysis. ChEMBL accumulates standardised bioactivity, molecule, target and drug data extracted from multiple sources (including literature). It contained 1,678,393 molecules with bioactivities defined as Ki, IC50, EC50 among others, and 11,224 unique targets for different species.

#### Kyoto Encyclopedia of Genes and Genomes (KEGG)

The KEGG pathway database (Release 94.1, May 1, 2020, https://www.kegg.jp) is a collection of manually drawn pathway maps representing the known molecular interactions, reactions and relations networks for several pathway categories such as the metabolism, cellular processes, genetic information processes, human diseases, or drug development [29]. The KEGG pathway was integrated into the drug-target library collected from ChEMBL.

#### Gene ontology (GO)

The Gene ontology (GO) resource (release 2020-05, http://geneontology.org) provides computational models of biological systems from many different organisms, from humans to bacteria, at the molecular level to pathways level. It can provide an annotation to the biological function and process of a protein. It contained more than 44,500 GO terms, 29,211 biological process terms, 11,113 molecular function terms and 4184 cellular component terms for  ~ 1.4 M of annotated gene products and 4593 Annotated species [30].

#### Human disease ontology (DO)

The DO resource (release 45, v2018-09-10, http://www.disease-ontology.org) provides a human-readable and machine-interpretable classification of biomedical data that are associated with human disease [30]. The DO resource includes 9069 DO identifiers (DOID) disease terms.

#### Morphological profiling

Morphological profiling data from 20,000 compounds were gathered from the Broad Bioimage Benchmark Collection (BBBC) using the BBBC022 dataset called “Human U2OS cells—compound-profiling Cell Painting experiment” [32] (information: https://data.broadinstitute.org/bbbc/BBBC022/). Basically, U2OS osteosarcoma cells were plated in multiwell plates, perturbed with the treatments to be tested, stained, fixed, and imaged on a high-throughput microscope. Then, an automated image analysis using CellProfiler (http://cellprofiler.org/) identified individual cells and measured morphological features on each of them in the aim to produce a cell profile [33]. In the end, the comparison of the cell profiles treated with different molecules (or experimental perturbations) allowed to suit different objectives such as identifying the phenotypic impact of chemical or genetic perturbations, grouping compounds and/or genes into functional pathways, and identifying signatures of disease [34]. In the BBBC022 dataset, there are 1779 morphological features measuring intensity, size, area shape, texture, entropy, correlation, granularity, angle between neighbours, etc. These parameters concern three “cell objects”: the cell, the cytoplasm and the nucleus. For our study, only the relevant information was kept. As each compound has been tested between 1 and 8 times, the average value of each feature for each compound was used. Features with a non-zero standard deviation and not correlated with each other (less than 95%) were kept in each of the three classes. Finally, we have extracted the data matching the compounds extracted from the ChEMBL database.

### Methods

#### Scaffold hunter

We used a software called ScaffoldHunter [35] to cut each molecule into different representative scaffolds and fragments as follow:

(i) Removing all terminal side chains preserving double bonds directly attached to a ring.

(ii) Removing one ring at a time using a set of deterministic rules in a stepwise fashion to keep the most characteristic “core structure” until only one ring is left.

**Fig. 1:**
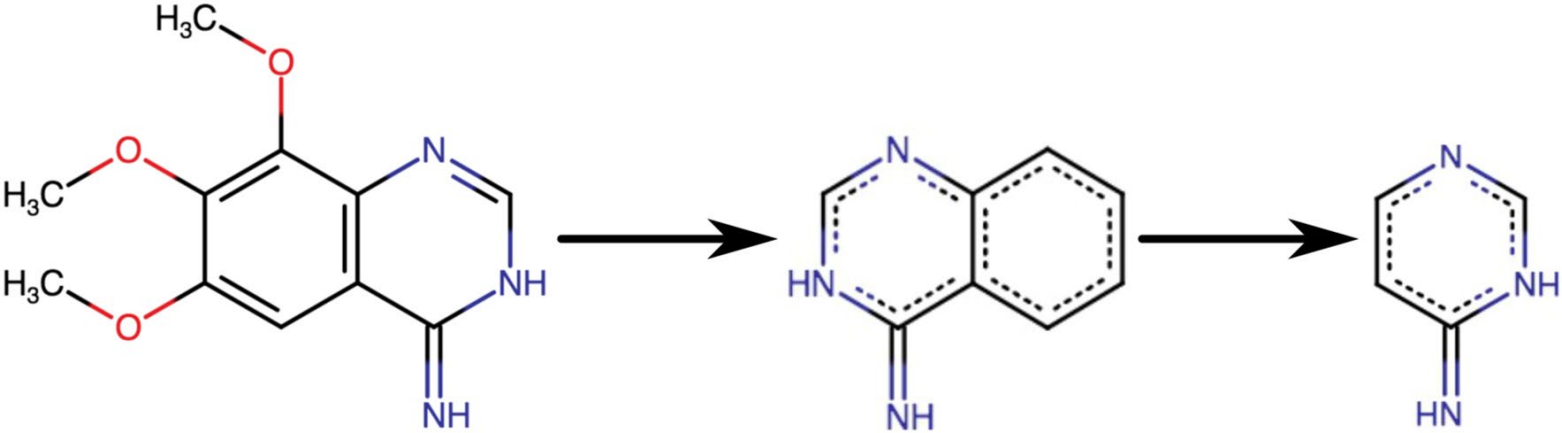
Illustration of the cutting process of Scaffold Hunter, from the whole molecule to one ring

Scaffolds are distributed in different levels based on their relationship distance from the molecule node (Fig. 1).

#### *Neo4J*^®^

The main tool used to create the graph database is Neo4j^®^ (https://neo4j.com/). It allows the integration of large scales of data from numerous sources. Its architecture is composed of nodes that represent a specific object (e.g., molecules, scaffolds, proteins, pathways, diseases…) linked by edges representing a relationship between two nodes (e.g., a scaffold being part of a molecule, a molecule targeting a protein, a target that acts in a pathway, etc.).

#### R package (cluster profiler, ggplot…)

R package cluster profiler (version 3.14.3) was used to calculate the GO enrichment and KEGG enrichment [36]. The R package DOSE (version 3.12.0) was used to perform the DO enrichment [37]. All the enrichment functions were used with the adjustment method “Bonferroni” and the p-value cutoff set at 0.1.

The R package org.Hs.eg.db [38] (version 3.10.0) was used to translate “EntrezID” [unique gene ID from the Entrez Gene database at the National Center for Biotechnology Information, (http://www.ncbi.nlm.nih.gov/gene)] to SYMBOL (Gene Name) and GO term.

#### Network pharmacology building

The heterogeneous sources of data were integrated into a network pharmacology database. First, we only selected compounds that have at least information on one bioassay (5,03,000 molecules) and integrated them in two main nodes of our network: “Molecule”, containing InchiKey and SMILES information and “CompoundName”, containing the chemical name and the database from which that name was extracted. We added 3 types of nodes related to assays: “Result” which mainly contains the value of the assay (from IC50, Ki…), linked to an “AssayParameter” providing the type of assay (IC50, Ki…) and unit of the value. The type of assay between A (ADME), B (Binding), F (Functional), U (Unassigned) and the confidence score defined by ChEMBL with a scale between 0: uncurated data and 9: direct target assigned were included. We integrated the “Target” node corresponding to protein targeted by the assays and only considered three species: human, rat and mouse. Then, we created a node “UniprotInter” (UI) which contains the generic ChEMBL name without species information and the added UniProt [39] (corresponding to the “Entry_name” in Uniprot).

The “UniprotInter” nodes were linked to a “ProteinClass” node extracted from the ChEMBL and containing information on the protein class to which a protein belongs. This classification schema has several levels (from 1 to 7) and goes from a specific classification (i.e., Metallo Protease M10A subfamily) to a general one (i.e., Enzyme). An example of this classification schema is illustrated in Additional file (Additional file 1: Figure S1).

For compounds present in the network and for which morphological profile is known, 3 nodes (“CellDesc”, “NuclDesc” and “CytoDesc”) including major features on these respective compartments (cell, nucleus and cytoplasm) were linked to the compound (CompoundName node).

The KEGG, DO and GO nodes are linked to the targets that are involved in the pathways and diseases respectively. As one target may act in several pathways or diseases, a single pathway and disease node can be linked to several targets.

#### Compound’s selection

For the compounds’ selection, only bioactive molecules with level 2 scaffolds and first-level protein classes were considered. It allows removing large series of molecules having too many analogues that can be kept with level 1 scaffolds and limit the association of a large set of molecules to general scaffolds such as benzene. Also, to limit promiscuous compounds, all scaffolds that were linked to more than 6 targets were removed.

As the “Target” information is regrouping 3 species, one target may be represented multiple times with only the species varying (e.g., 5HT1A_HUMAN and 5HT1A_RAT). To remedy this issue, we use the “UniprotInter” (UI) node that does not take species into account, so the information is not redundant.

Then, a binary matrix that annotated the bioactivity profile for each scaffold (in rows) with all the targets (in columns) was created. Scaffolds belonging to an active compound with a bioactivity for a target was noted as 1, 0 otherwise. Based on this matrix, hierarchical clustering was performed to separate the scaffolds into clusters.

We decided to select one scaffold per cluster using the following principle:

- The scaffold with the lowest distance, based on a distance matrix using the dist function in R with the binary method, equivalent to Jaccard/Tanimoto indices.
- If there were scaffolds with the same distance, we selected them based on the number of targets they hit, the highest being prioritised.
- Finally, we chose the scaffold that is linked to the highest number of molecules.
  If all of these criteria were not able to filter one scaffold by cluster, we considered the scaffolds to be similar and took one among the ones remaining.
  Once all the scaffolds were selected, we extracted all active molecules linked to them and performed a multiobjective Pareto optimisation [40] using Pipeline Pilot to select 5000 molecules that will represent the chemogenomic space present in ChEMBL.
  Similarly, to the scaffold selection, the compound selection by Pareto was based on 3 criteria:
- Prioritise molecules with the most targets to maximise the different biological profiles.
- Prioritise molecules to maximise the number of scaffolds selected.
- Prioritise molecules to maximise the average number of times a target is hit.

The Pareto method uses a genetic algorithm to generate the best subsets possible. The considered parameters were several subsets created up to 1000, a subset size of 5000 compounds and 600 iterations. The mutation rate parameter was unchanged.

## Results

### Network pharmacology development

**Fig. 2:**
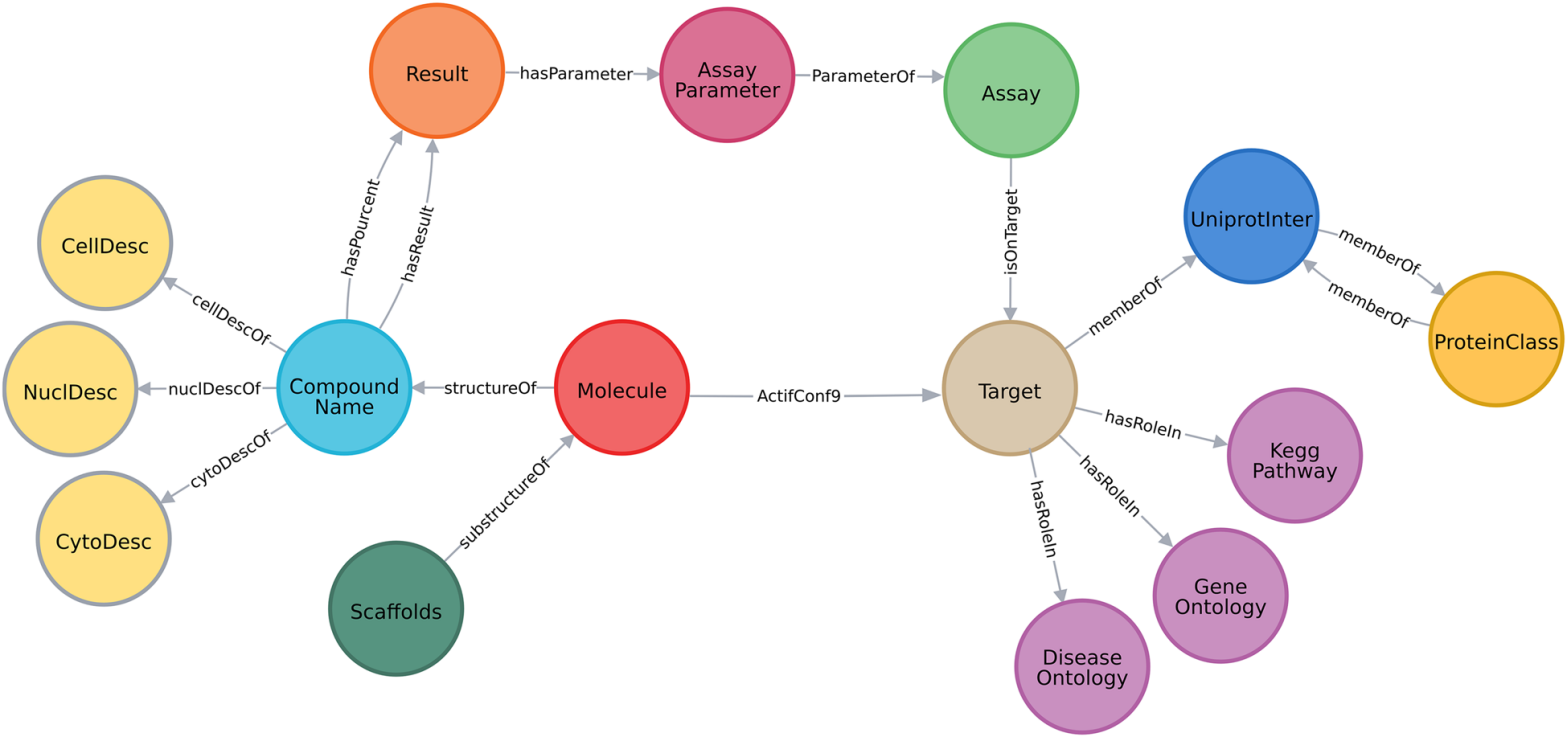
Overview of the database, coloured by the type of node, the arrows corresponding to the type of relationship between the nodes. The CellDesc, the NuclDesc and the CytoDesc represent features related to the compartment Nucleus, Cytoplasm and Cell observed on the U2OS cell line

A representation of the final graph database developed with Neo4J is shown in Fig. 2. Globally, 1,61,468 molecules that have a Ki/IC50 activity below 1 μM, a confidence score of 9 among bioassays of type B and bioactive in mouse, rat and human were integrated into the network. This ensemble of compounds modulates 1975 targets which will be considered for further filtering steps. A direct link between the node “Molecule” and “Target” called actifConf9 was created to facilitate the database manipulation.

**Fig. 3:**
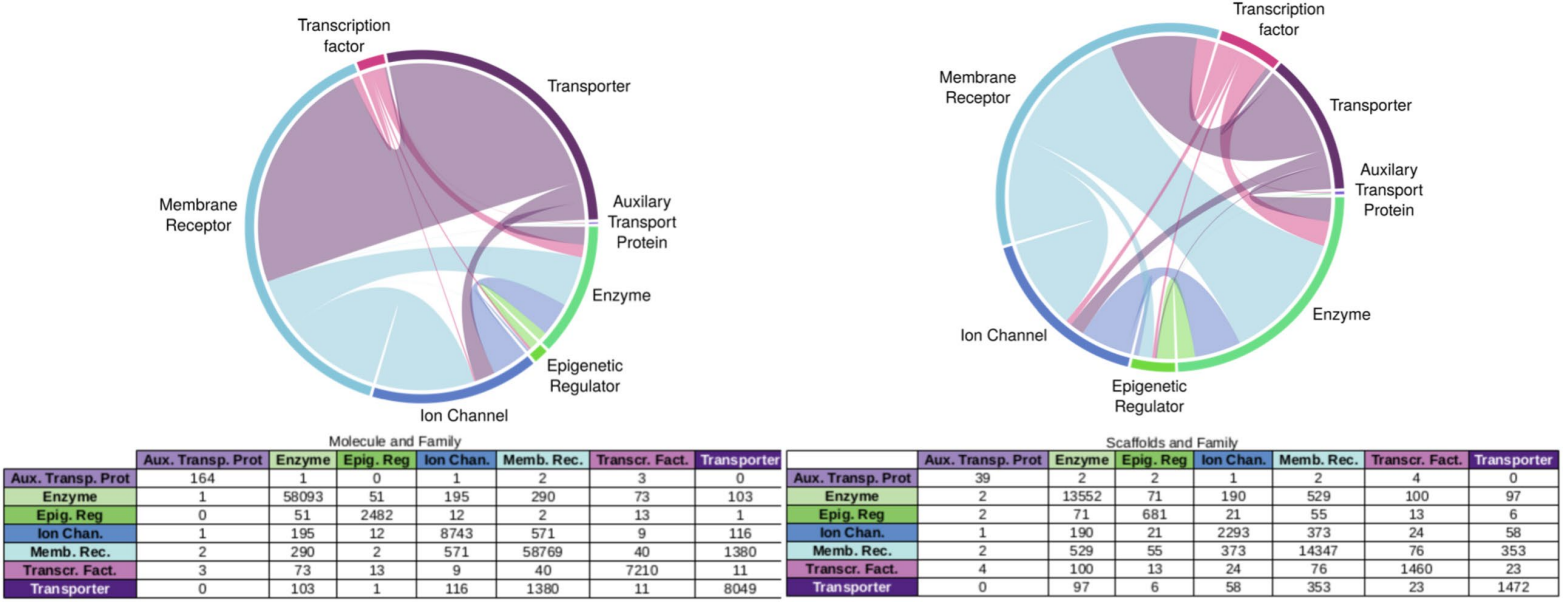
Chord diagram and distribution of **A** bioactive molecules and **B** scaffolds

From this set of bioactive compounds, 1,13,853 distinct scaffolds were generated and integrated into the network. For the protein classes, ChEMBL has defined 1073 distinct classes distributed in 7 main protein families. They represent the main area of drug discovery investigation, notably the membrane receptor [with the G protein-coupled receptors (GPCR)] and the enzyme. Only the protein classes at level 1 protein classes directly connected to a UniprotInter node were considered in this study ending up with 363 protein classes. The distribution of molecules and scaffolds into the 7 families are depicted in Fig. 3. Number of molecules (and scaffolds) that have been reported active on several protein families are also depicted in chord diagrams (Fig. 3). We noticed that among the molecules active on Transporter, many of them (1380 molecules) are also active on membrane receptors. In opposite only few molecules active on Auxiliary Transport Protein have been reported to be active on another protein family.

From the pharmacology network, several information can be obtained such as multiple targets profile associated with a compound and its scaffold. For example, crizotinib, an inhibitor of tyrosine kinase receptors used for the treatment of non-small lung cancer targeted several proteins that belong to different protein classes but all membered of one main protein family, kinase (Additional file 1: Figure S2). Interestingly, looking through the network, the number of molecules sharing the same scaffolds with crizotinib can be collected. This set of molecules could be suggested to have activities on these tyrosine kinase receptors. Similarly, potential new bioactivities not observed in previous studies could be proposed to crizotinib based on scaffold similarity with bioactive molecules.

**Fig. 4:**
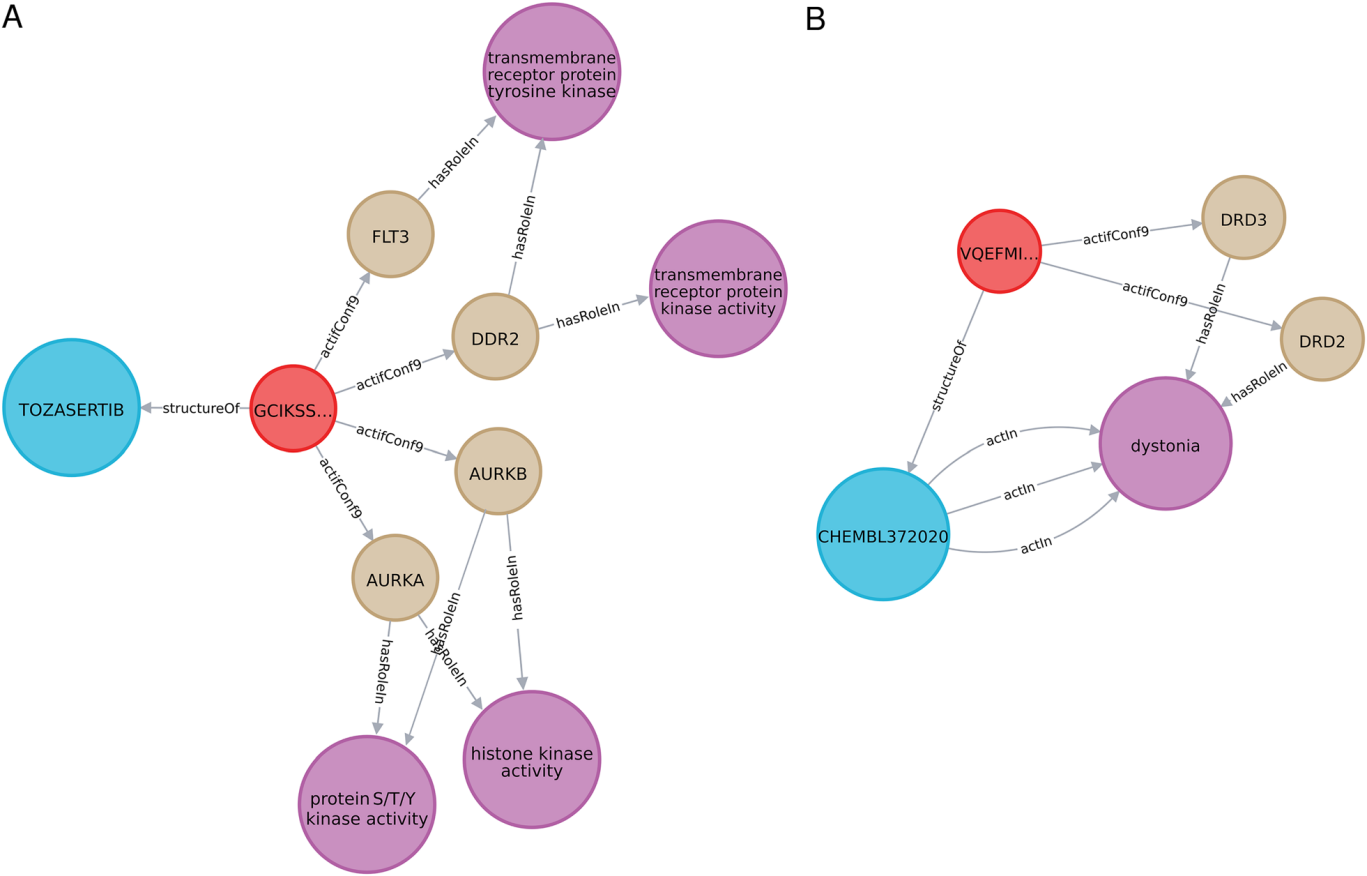
Example of pathway enrichment for the **A** tozasertib molecule involved in 4 GO and **B** Chembl372020 molecule [(S)-7-Dipropylamino-5,6,7,8-tetrahydro-indolizine-3- carbonitrile] involved in DO “dystonia”

Furthermore, based on the drug-targets network, it was possible to include pathways and diseases information allowing us to highlight known links between chemicals, proteins, pathways and diseases. In this network, we were able to add 766 GO terms, 301 KEGG pathway terms and 562 diseases ontology terms (DO) and performed enrichment analyses. For each compound linked to at least two proteins that are involved in the same pathway, a p value (adjusted according to the number of genes involved) was computed. It allowed to directly link compounds to pathways and to determine pathways that are statistically enriched in a protein’s list. For example, the tozasertib molecule (pan-Aurora kinase inhibitor, anticancer treatment) is linked to 4 proteins: FLT3 (Fms-like tyrosine kinase 3), DDR2 (Discoidin domain receptor tyrosine kinase 2), AURKB (Aurora kinase B) and AURKA (Aurora kinase A) (Fig. 4A) in our network. Two of these targets (FLT3 and DDR2) are involved in the same gene ontology (GO) term “transmembrane receptor protein tyrosine kinase” (GO:0004714). The enrichment for this GO term showed a calculated p value of 2.54e-24, meaning that the tozasertib has a significant influence on the transmembrane receptor protein tyrosine kinase activity. Interestingly, the AURKA and AURKB genes are also involved in kinase activities (histone kinase activity and protein S/T/Ykinase activity) whose activations are necessary for cell division processes in the regulation and control of mitosis. All of these proteins play an important role in a wide range of cancers and it explains the interest of tozasertib as an anticancer treatment. As a second example, the molecule Chembl372020 [(S)-7-Dipropylamino-5,6,7,8-tetrahydro-indolizine-3-carbonitrile] is linked to two targets/genes, DRD3 (dopamine receptor D3) and DRD2 (Dopamine receptor D2), both involved in dystonia. The calculated p value enrichment for the DO term (represented in Fig. 4B by the relation arrows “actIn”) is 8.42e-05. It means that the molecule is significantly involved in dystonia through DRD3 and DRD2 genes.

### Morphological profile integration

Finally, we integrated the morphological profiles for compounds in common between the ChEMBL and the BBBC dataset. We found 2473 compounds common to both datasets. It means that for this set of compounds, proteins are annotated and can be suggested to the morphological perturbations observed in the U20S cell line. Morphological features are included in the network according to the 3 cellular components described in Cell Painting: the nucleus, the cytoplasm and the whole cell itself (respectively named “nucl.”, “cyto.” and “cell”). This phenotypic information could highlight links between the target compartment and the phenotypic variations associated with the molecule. Among others, features may be a measure of the mean radius of the cytoplasm area shape (“Cytoplasm_AreaShape_MeanRadius”), the location of the centre of the cell according to the X-axis (“Cells_Location_Center_X”) or the entropy in the nucleus of the cell (“Nuclei_Texture_Entropy”).

A features selection was applied for features concerning the same cellular component. Among the 1779 features, only 767 were kept: 250 for cell, 261 for cyto and 256 for nucl respectively. Overall, a relation between a bioactive molecule on specific proteins and morphological perturbation can be suggested. For example, ciglitazone, a thiazolidonedione with potential interest in ovarian hyperstimulation syndrome or as an anti-hyperglycemic agent is a selective agonist to the nuclear receptor PPARy (Peroxisome proliferator-activated receptor gamma) and shows morphological perturbations for different features i.e., “Cytoplasm_Correlation_Manders_DNA_ER”, “Cytoplasm_Correlation_Manders_RNA_ER”' or “Cells_Correlation_Manders_Mito_ER”. So, this analysis could suggest a relation between the activation of PPARy and the morphological disturbance of some compartments in cells.

### Chemogenomics library development

**Fig. 5:**
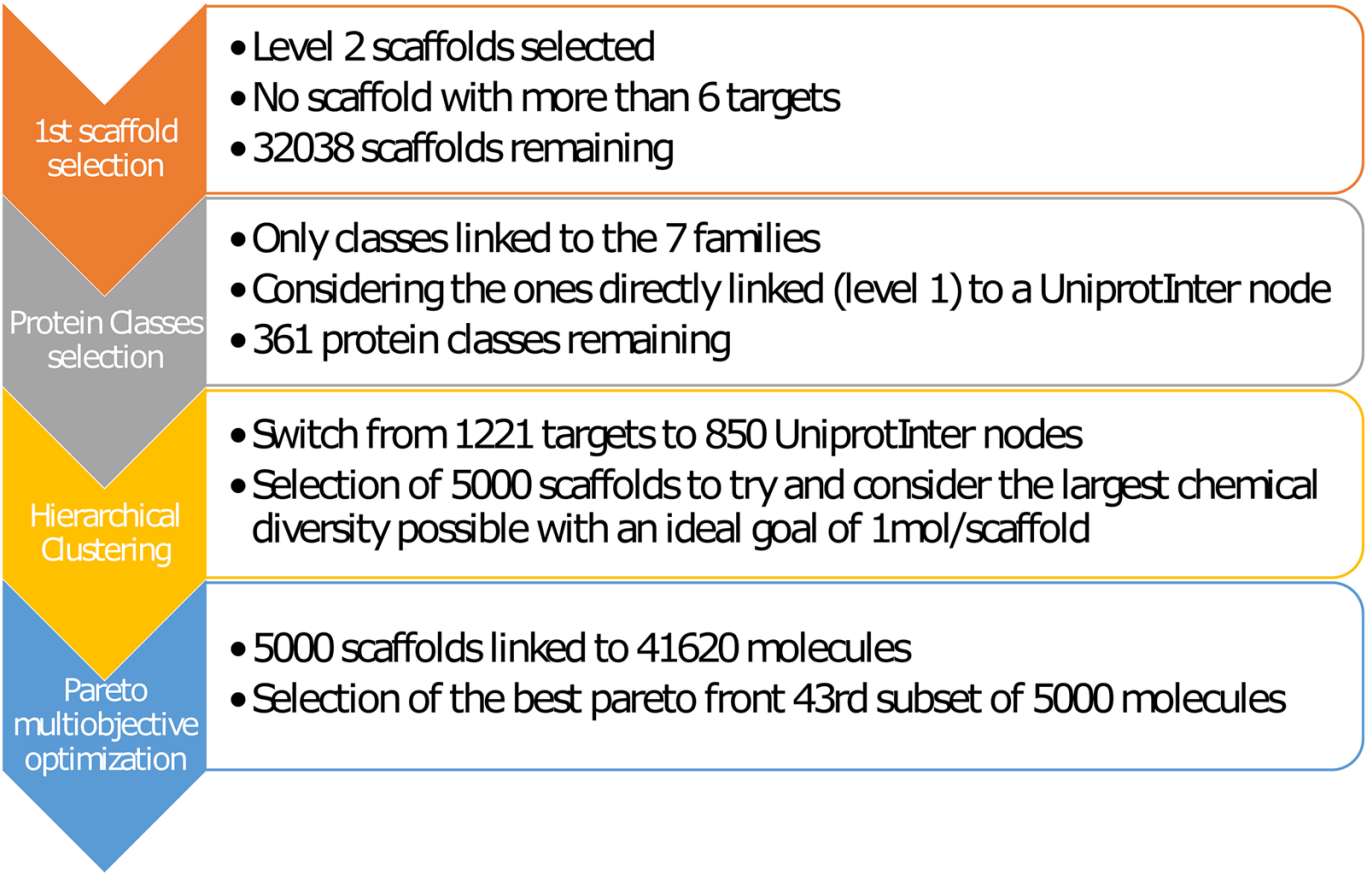
Workflow of the 5000 molecules selection

Based on our graph database, we decided to develop a chemogenomic library of 5000 molecules that would cover the chemogenomic space and could be used for phenotypic screening. A workflow of the protocol is shown in Fig. 5.

In the first step, from the set of bioactive molecules, we selected sub-scaffolds at level 2. Such selection allowed to remove too specific scaffolds of a molecule observed at level 1, but still capturing selectivity of molecules associated with some proteins. The main objective is to avoid a general scaffold (i.e., only a ring) that would not be specific enough to discriminate between molecules when trying to select active ones for a target. Then, to limit promiscuity, all scaffolds that were linked to more than 6 targets were removed, (being the beginning of the curves' elbow in Additional file 1: Figure S3), retaining 32,038 scaffolds.

In a second step, we focused on the protein’s space. From the 7 main protein’s classes defined in ChEMBL, only the first level protein classes were selected and connected to 1221 protein’s targets resulting in 363 protein classes. This left us with 32,038 scaffolds linked to 1221 targets belonging to 363 Protein Classes. On average, there were 3.4 molecules/scaffold and 1.5 targets/scaffold.

In our network pharmacology, the 1221 targets correspond to 850 UniprotInter nodes (UI) i.e., proteins having a unique function, independently of the species. From there, the third step consisted of establishing a set of 5000 molecules that cover as much as possible the 850 UI. We decided to select 5000 scaffolds, to have a high diversity of molecules covering the protein’s space. To do that we developed a hierarchical clustering that allowed us to select 5000 scaffolds linked to 41,620 molecules and hitting 850 UI. Then, we performed a Pareto multiobjective optimisation which selected the best subsets of 5000 molecules satisfying the criteria defined in the method section.

The Pareto optimisation created multiple “fronts” which correspond to a dataset containing multiple subsets of 5000 molecules. They had the same range of results concerning the criteria and we decided to select the one maximising both the biological profile and the number of scaffolds. As such the 43rd out of 170 subsets from the 1st front matched those criteria and was chosen to represent the 5000 compounds (Additional file 1: Figure S4).

We figured out that by selecting protein classes at level one, some proteins (94 proteins) were not targeted by one of the 5000 compounds or their scaffolds. This is due to the ChEMBL proteins classification schema for which some proteins were not associated with one of the 7 main families. Therefore, in a final step, to cover the maximum of the chemogenomic space, bioactive compounds to missing proteins and capturing most of the molecules through their scaffold were included in the prior set of molecules. Overall, we obtained a library of 5100 molecules with a high diversity of scaffold targeting all bioactive proteins in ChEMBL and that could be used for phenotypic screening. We can observe in this library that on average, there are a little more than two active compounds per protein i.e., with a Ki or IC_50_ lower than 1 μM. Many compounds are active on several proteins (see Additional file 1: Figure S5) which allow associating several scaffolds to a specific protein but also to determine the promiscuity of proteins with scaffolds that could be of interest in the design of drugs acting on multiple targets or disease pathways i.e., polypharmacology.

Interestingly, only a few chemicals from this library also had information on the Cell painting data and around 10% of the compounds in the phenotypic data is also present in ChEMBL. In addition, many of these compounds did not pass the confidence score (score of 9) applied in ChEMBL and a bioactivity threshold (< 1 μM) that allow selecting highly active compounds. It means that only a few chemicals are shared between the two databases. Nevertheless, for these chemicals a relation between their morphological profiles and molecular mechanisms could be proposed.

### Discussion

With the aim to relate the modulation of the protein’s function by chemicals to some phenotype variations, we created a system pharmacology network, integrating chemical-protein-pathways and phenotypic screening from two different sources, disease ontology and morphological features of cells. The representation of the molecules into scaffold facilitates the recognition of chemotypes i.e., chemical patterns (opioid, benzodiazepine…) associated with specific proteins, the diversity of scaffolds linked to a protein and the diversity of proteins targeted by a series of molecules with a unique scaffold. The incorporation of phenotypic data allows us to go one step further and to assist in the target deconvolution of phenotypic assays. Although high content imaging analysis allow to observe and to measure the morphological disturbance of a cell by a chemical, such technology do not give information about the molecular mechanism that underlies the cell perturbation. The integration of chemical-protein activity from ChEMBL with chemical-morphological profile from Cell Painting, can help to identify proteins that could explain the morphological change of a cell by a chemical and so the potential phenotypic and/or disease impact. The drug-targets-pathways-diseases relationships might help in the investigation of repurposing drugs or a combination of bioactive drugs on two complementary proteins involved in the same pathway. The system pharmacology network is not fully accomplished and phenotypic outcomes could be caused by some targets not yet determined for a compound. Other databases could be integrated. Among them, PubChem [41], ChemProt, DrugCentral [42] databases would be useful to enrich drug-target interactions. Furthermore, with microarray and next-generation sequencing technology, deregulation of genes and pathways caused by a compound in specific conditions (dose, time, cell type, organ, species) like for example in LINCS [43] would be beneficial for obtaining a more comprehensive chemogenomic network. Several initiatives have been developed to identify modes of action of bioactive compounds based on transcriptomics data to suggest new therapeutic indications for a variety of diseases [44, 45]. For example, Iskar et al. combined drug-target information and gene expression profiles after drug treatment to identify the deregulation of new drug-target interactions that could explain the repurposing of drugs or potential side effects associated with them [46]. It is important to notice that the scaffold composition is highly dependent on screening libraries considered and methods used to generate scaffolds [47, 48]. Recently, the implementation of scaffold network has been introduced as a powerful method to navigate and to analyze large screening data sets and could be an alternative to the scaffold selection used in our study [49, 50]. Also, in addition to scaffolds that can help to recognize certain chemotypes, other methods based on activity cliffs could be interesting to integrate as it consists of interpreting a set of structurally similar compounds with a large difference in potency against their target [51].

Overall, our systems pharmacology network captures a large ensemble of drug-target interactions with high confidence and based on a state-of-the-art NOSQL graphics database (Neo4J) facilitating the manipulation of large sets of data in a fast and efficient manner. The integration of biological data such as pathways, diseases and phenotypic screening allows to study the effect of a molecule not only at the molecular level but also in more complex layers of a systems biology and can reveal novel repurposing and synergistic therapeutic opportunities or drug safety issues.

Once the systems pharmacology network was developed, we decided to develop a chemical library limited to 5000 molecules that could be of interest in phenotypic drug discovery campaigns. Several aspects have been considered in the development of the library such as (i) accuracy about drug’s bioactivity (ii) diversity of molecular scaffolds (iii) diversity of targets and target family across the human proteome (iv) diversity of pathways perturbations and diseases associated with chemicals.

Eventually, we obtained a library of 5100 compounds targeting a large ensemble of the proteome i.e. 1234 proteins corresponding to 944 UI (Additional file 2). Compared to GSK and Pfizer libraries which are dominated by kinase, GPCR (Pfizer also includes ion channels), our chemical library is more diverse as it contains transcription factor, enzyme and epigenetic receptors among others. The number of 5000 compounds was chosen based on the fact that it converges to the size of libraries reported by pharmaceutical compagnies (~ 3000 for Pfizer and  ~ 6000 for GSK libraries respectively)[52]. Our library certainly not covers the complete chemogenomic space but it is more affordable compared to a full HTS, still encompassing a large set of chemical-protein interactions represented in ChEMBL, that is suitable for a hit identification study in early drug discovery program.

The diversity of scaffolds and biological profiles obtained through the Pareto selection give also a much more comprehensive representation of the proteome. Further selections of compounds impacting the genome, and thus other targets, could be performed using other technologies from genomic screening (si/shRNA, CRISPR-Cas9, RNAi, transcriptomics).

Based on this study, we identified 2473 chemical-target interactions from ChEMBL with morphological profiles from Cell Painting. At the scaffold level, common chemotype associating scaffold-proteins and morphological profiling can be suggested. The fact that our chemical library is essentially based on compounds with pharmacological interest will probably have a better merit in deciphering pharmacological mechanisms with disease phenotypic screening. Including some compounds known to generate a broad range of toxic mechanisms would be necessary to predict cellular phenotypic profiles with molecular perturbations.

## Conclusion

The developed systems pharmacology network is an interesting tool that can be used in drug recommendation and repurposing. The integration of pathways and phenotypic data allows linking molecular mechanisms to disease pharmacological compounds. Additional data such as high-throughput transcriptomic would be interesting to incorporate in such a network to get insights into the genome-scale perturbation of a compound. Expanding on our previous efforts with a combination of proteome and transcriptome modulations by compounds and linking these data with phenotypic screening would pave the way in phenotypic drug discovery. Furthermore, optimization of a chemical library that would encompass the information coming from these new chemical biology technologies would facilitate the identification of molecular mechanisms to phenotype and the discovery of novel pharmacological entities.

## Supplementary Information

**Additional file 1: Figure S1.** The “proteinClass” node (in yellow) Serine protease is a level 1 protein class, and the node Protease is a level 2 protein class for the “UniprotInter” node Serine protease hepsin (colored in blue). They are linked by a relationship member of which indicates their belonging to a specific family. **Figure S2. **Example of network representation with crizotinib. 1 molecule, multiple targets hit in multiple protein classes in one main family. **Figure S3.** Repartition of the number of targets for each scaffold, with the repartition curve in red. **Figure S4.** Overview of the 43th pareto front selection between the maximization of the different biological profiles (x axis) and the average number of times a UI is hit (y axis). Each iteration is of a different colour, each point equal 1 out of the 5000 molecules. **Figure S5.** Bar chart of the number of UI targeted by the final selection of molecules.

**Additional file 2: Table S1.** List of 5100 compounds bioactives on proteins. The compounds are encoded with a ChEMBLID, InChiKey and SMILES code.

## Data Availability

The chemical library, with ChEMBL ID, SMILES, InchiKey and bioactive proteins associated, is available on Additional file. The code to reproduce the work is available on GitHub at this link: bit.ly/3Bs1w3u.
