# Supplementary material for: Development of a chemogenomics library for phenotypic screening
Source: J Cheminform. 2021 Nov 24;13:91. doi: 10.1186/s13321-021-00569-1 (PMC8611952; doi:10.1186/s13321-021-00569-1)
Supplement: Supplementary file 1 — Additional file 1: Figure S1. The “proteinClass” node (in yellow) Serine protease is a level 1 protein class, and the node Protease is a level 2 protein class for the “UniprotInter” node Serine protease hepsin (colored in blue). They are linked by a relationship member of which indicates their belonging to a specific family. Figure S2. Example of network representation with crizotinib. 1 molecule, multiple targets hit in multiple protein classes in one main family. Figure S3. Repartition of the number of targets for each scaffold, with the repartition curve in red. Figure S4. Overview of the 43th pareto front selection between the maximization of the different biological profiles (x axis) and the average number of times a UI is hit (y axis). Each iteration is of a different colour, each point equal 1 out of the 5000 molecules. Figure S5. Bar chart of the number of UI targeted by the final selection of molecules. [file 13321_2021_569_MOESM1_ESM.pdf]

Supplementary Data

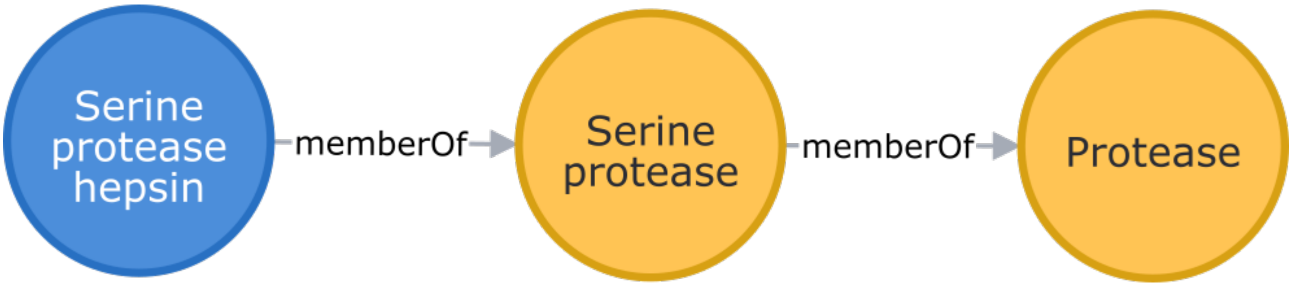

Figure S1: The "proteinClass" node (in yellow) Serine protease is a level 1 protein class, and the node Protease is a level 2 protein class for the "UniprotInter" node Serine protease hepsin (colored in blue). They are linked by a relationship *memberOf* which indicates their belonging to a specific family.

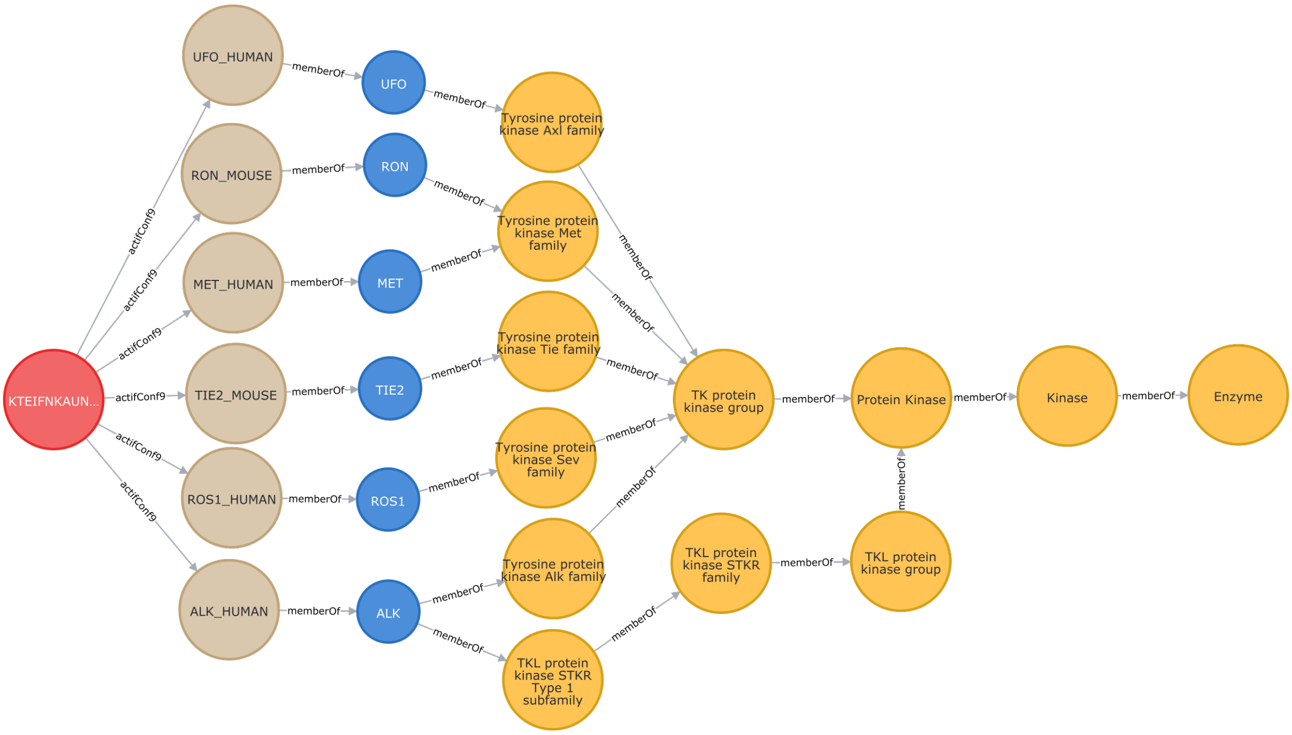

Figure S2: Example of network representation with crizotinib. 1 molecule, multiple targets hit in multiple protein classes in one main family.

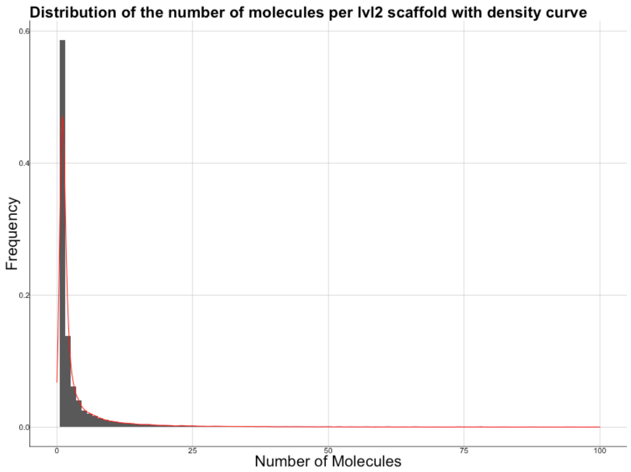

Figure S3: Repartition of the number of targets for each scaffold, with the repartition curve in red.

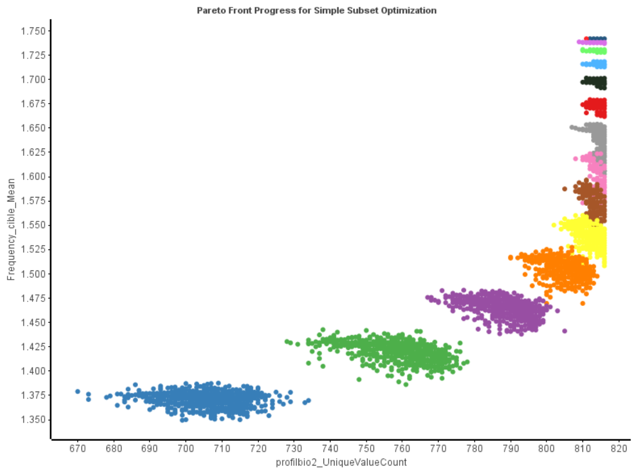

Figure S4: Overview of the 43th pareto front selection between the maximization of the different biological profiles (x axis) and the average number of times a UI is hit (y axis). Each iteration is of a different colour, each point equal 1 out of the 5000 molecules.

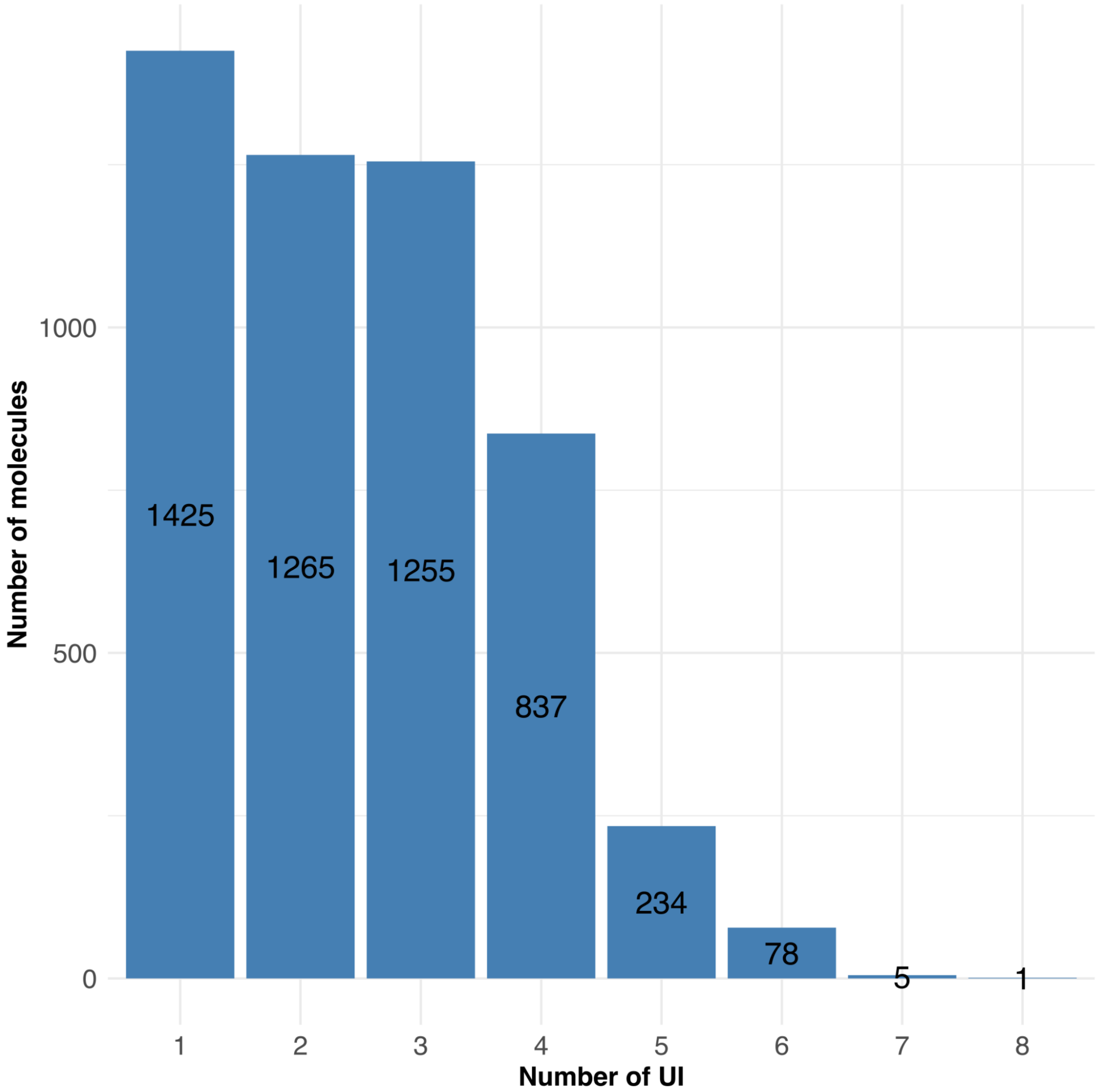

Figure S5: Bar chart of the number of UI targeted by the final selection of molecules
